# Spatial navigational strategies correlate with gray matter in the hippocampus of healthy older adults tested in a virtual maze

**DOI:** 10.3389/fnagi.2013.00001

**Published:** 2013-02-20

**Authors:** Kyoko Konishi, Véronique D. Bohbot

**Affiliations:** Department of Psychiatry, Douglas Institute, McGill UniversityVerdun, QC, Canada

**Keywords:** aging, radial maze, spatial memory, hippocampus, navigation

## Abstract

Healthy young adults use different strategies when navigating in a virtual maze. Spatial strategies involve using environmental landmarks while response strategies involve executing a series of movements from specific stimuli. Neuroimaging studies previously confirmed that people who use spatial strategies show increased activity and gray matter in the hippocampus, while those who use response strategies show increased activity and gray matter in caudate nucleus (Iaria et al., 2003; Bohbot et al., 2007). A growing number of studies report that cognitive decline that occurs with normal aging is correlated with a decrease in volume of the hippocampus. Here, we used voxel-based morphometry (VBM) to examine whether spatial strategies in aging are correlated with greater gray matter in the hippocampus, as found in our previous study with healthy young participants. Forty-five healthy older adults were tested on a virtual navigation task that allows spatial and response strategies. All participants learn the task to criterion after which a special “probe” trial that assesses spatial and response strategies is given. Results show that spontaneous spatial memory strategies, and not performance on the navigation task, positively correlate with gray matter in the hippocampus. Since numerous studies have shown that a decrease in the volume of the hippocampus correlates with cognitive deficits during normal aging and increases the risks of ensuing dementia, the current results suggest that older people who use their spatial memory strategies in their everyday lives may have increased gray matter in the hippocampus and enhance their probability of healthy and successful aging.

## Introduction

Multiple studies have demonstrated that age impairs spatial memory in both humans and non-human species (Barnes et al., 1980; Marighetto et al., 1999; Driscoll et al., 2003; Wood and Dudchenko, 2003; Moffat et al., 2006; Antonova et al., 2009; Iaria et al., 2009). Older adults report to having more navigation problems with increasing age and admit to avoiding traveling unfamiliar routes (Burns, 1999). Furthermore, “getting lost” behavior and impairment in spatial memory are early symptoms of cognitive impairment and Alzheimer's disease (Klein et al., 1999; deIpolyi et al., 2007; Hort et al., 2007).

When navigating in a virtual environment, studies showed that participants spontaneously use one of two navigational strategies (Packard and McGaugh, 1996; Iaria et al., 2003). Spatial memory, or the “spatial strategy,” is one of two navigation strategies that can be used when going places (Packard et al., 1989; Packard and McGaugh, 1996; Iaria et al., 2003; Bohbot et al., 2007). It involves navigating within an environment by forming relationships between different landmarks in the environment and orientating oneself in relation to those landmarks. This process of forming associations between landmarks leads to the development of cognitive maps, i.e., mental representations of one's environment. Knowledge of the relationships between landmarks is characterized with flexibility, allowing, for example, one to derive a direct path to a target destination when navigating in a town (O'Keefe and Nadel, 1978). The spatial strategy is subserved by the hippocampus (O'Keefe and Nadel, 1978; Morris et al., 1982; Abrahams et al., 1997; Bohbot et al., 1998). In contrast, the “response strategy” involves learning a series of stimulus-response associations such as a pattern of left and right turns from a given starting position. This strategy is inflexible in the sense that it does not allow deriving a direct path to a target location (O'Keefe and Nadel, 1978). The response strategy relies on the striatum, which includes the caudate nucleus in humans (Packard et al., 1989; Packard and Knowlton, 2002; White and McDonald, 2002).

In a previous study (Bohbot et al., 2007), we tested healthy young adult participants on a virtual navigation task that can be solved using either a spatial or response strategy. We found that spatial learning positively correlated with gray matter in the hippocampus, while response learning positively correlated with caudate nucleus gray matter. Furthermore, an inverse relationship was found between gray matter in the hippocampus and caudate nucleus. In other words, those who had more gray matter in the hippocampus had less gray matter in the caudate nucleus and vice versa. In addition, participants' peak hippocampal values correlated with gray matter in a network of regions anatomically connected to the hippocampus, such as the perirhinal, entorhinal and parahippocampal cortices, the orbitofrontal cortex, and the amygdala. There was also a significant correlation with gray matter in the contralateral hippocampus.

Several studies have examined the neural correlates of spatial memory in older adults. Using manual segmentation, Chen et al. (2010) and Head and Isom (2010) found that performance on a virtual spatial memory task correlated with total volume of the hippocampus. Head and Isom (2010) additionally found a positive correlation between caudate nucleus volume and performance on a virtual route learning task, a task known to utilize response strategies.

In the present study, we investigated whether performance on our navigation task that distinguishes between spontaneous spatial and response strategies correlated with gray matter in the hippocampus and caudate nucleus in healthy older adults, using the technique of voxel-based morphometry (VBM). We hypothesized that older adults who use spatial strategies would have more gray matter in the hippocampus while those who use response strategies would have more gray matter in the caudate nucleus. Furthermore, similar to results reported in Bohbot et al. (2007), we hypothesized that those with more gray matter in the hippocampus would have more gray matter in the associated network of neuroanatomically connected regions. Since gray matter in the hippocampus is associated with healthy cognition in aging, the variability in navigational strategies could potentially help distinguish a healthy older adult from an older adult at risk for cognitive deficits. Identifying such risk factors can have a profound impact on the well-being of older adults. This is especially relevant considering the current international demographics showing a growing aging population. By the year 2050, 16% of the world population will be over the age of 65. In the US, 20% of the population is expected to be over the age 65 by 2050 and in Japan, 38% of the population is expected to be over 65 by 2050. Consequently, the current paper may bring novel insights into healthy and unhealthy aging that can help define early detection methods in view of early intervention to promote healthy aging.

## Materials and methods

### Participants

Forty-five healthy older adults (mean age = 64.38 ± 4.0; 23 women and 22 men) participated in the study. All participants were right-handed and had normal or corrected vision. All participants were screened for any history of neurological or psychiatric disorders, alcohol, or drug abuse using a pre-screening questionnaire. All participants underwent a neuropsychological assessment to control for dementia, depression, and mild cognitive impairment (Table 1). Informed consent was obtained from all participants in accordance with the guidelines of the local ethics committee. The study was approved by the Research Ethics Board at the Douglas Mental Health University Institute and the Montreal Neurological Institute.

**Table 1:** **Older adult participant demographics and test means [SEM]**.

**PARTICIPANT CHARACTERISTICS**	
*N*	45
Women:Men	23:22
Age (years)	64.38 ± 4.05
IQ	107.5 ± 13.12
Education (years)	15.48 ± 2.45
Handedness	Right
**NEUROPSYCHOLOGICAL TEST SCORES**	
MMSE	29.21 ± 0.91
MoCA	27.48 ± 1.65

### Behavioral tasks

#### Concurrent spatial discrimination learning task

**Figure 1:**
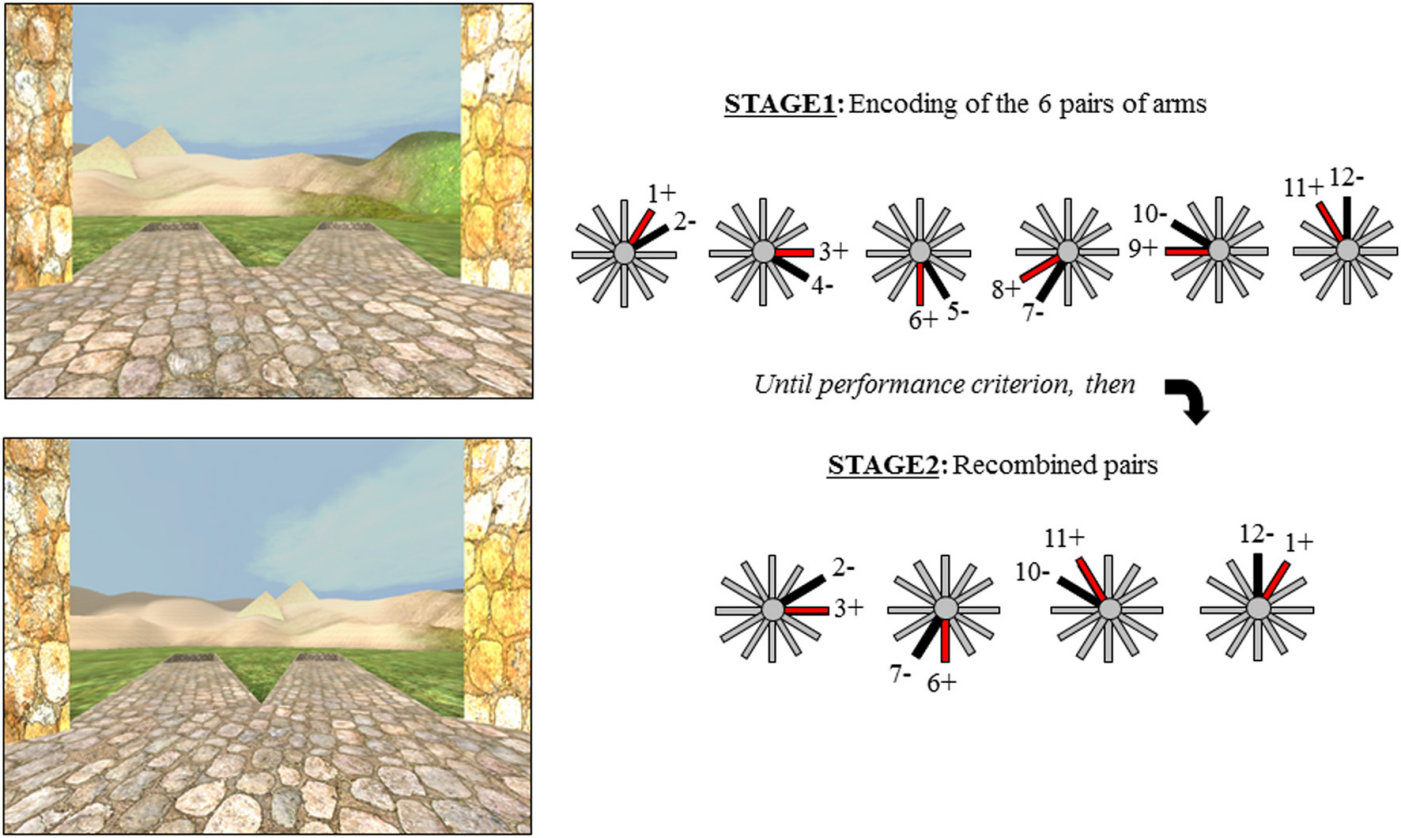
**The two stages of the virtual radial maze with a schematic representation of the behavioral paradigm.** Example of a pair of arms presented during encoding (Stage 1) and a recombined pair during Stage 2.

The Concurrent Spatial Discrimination Learning Task (CSDLT) was created using the editor program of Unreal Tournament 2003 (Epic Games, Raleigh, NC). The task is situated in a radial maze surrounded by an enriched environment made up of a landscape and proximal and distal landmarks such as plants and hills (Figure 1). It consists of a center platform from which branch out 12 pathways. At the end of each pathway, there is a set of stairs that leads to a small pit where, in some of the arms, an object is located. The 12 arms of the maze are divided up into six adjacent pairs of arms. Within each pair of arms, one arm always contains an object while the other is always empty. During the experimental encoding phase (Stage 1), participants are repeatedly presented with the six pairs of arms in a pseudo-random order. They are asked to learn which arm contains an object within each pair of arms and to go down that arm to retrieve the object. Upon descending the stairs at the end of the pathway and entering the pit, participants are automatically brought back to the center platform and presented with the next pair of arms. The number of correct arms the participant visits within each trial is measured as choice accuracy. One trial consists of the presentation of all six pairs of arms. Participants are trained until a choice accuracy criterion of 11/12 is reached within two consecutive trials. A minimum of six trials is administered to all participants.

Upon reaching criterion, participants proceed to a test phase aimed as dissociating the strategies used during the encoding phase of the task. The test or probe phase (Stage 2), is also called the “recombined pairs” condition because, during this phase, the arms presented to the participants are rearranged into novel pairs; however, the reward contingency remains the same. In other words, during the recombined pairs condition, the objects are placed at the end of the same arms as during the encoding phase. Four pairs of recombined arms are presented twice in a pseudo-random order. Only four recombined pairs allowed for the presentation of adjacent arms with only one arm containing an object. The recombined pairs condition was designed in such a way that successful performance on this stage requires that individuals be flexible at using the information they learned during the encoding phase of the task, evidenced by the fact that they know the spatial relationship between the object and environmental landmarks (i.e., the spatial strategy). In other words, when the pairs of arms that are presented are recombined, participants who know the relationship between the target objects and landmarks are capable of discerning the target arm from the non-target arm. In contrast, people who did not show flexibility during the recombined pairs have acquired the task by using a response strategy, i.e., “when I see the hills, take the arm to the left.” In this case, since the pairs of presented arms were rearranged, “when I see the hills, take the arm to the left” in this recombined pairs stage is not the same arm as in the encoding stage. Thus, the recombined pairs stage allows us to distinguish between participants who are flexible and used a spatial strategy and those who are inflexible and used a response strategy in an objective fashion.

#### Rey auditory verbal learning test

The Rey Auditory Verbal Learning Test (RAVLT) is a standard neuropsychological memory test (Lezak, 1995). A list of 15 words (list A) is read for five trials and after each trial the participant is asked to verbally recall as many words as they can remember. Next, an interference trial is provided whereby a different list of 15 words (list B) is read to the participant and again the participant is asked to recall as many words as they can remember. Following this interference trial, the participant is asked to recall as many words as they can from list A. Finally, after a 30-min delay, the participant is again asked to remember as many words as they can from list A. Performance was assessed with the number of words recalled after interference (AI) and after the 30-min delay (delayed recall).

#### Rey-osterrieth complex figure

In the Rey-Osterrieth Complex Figure task (RO) (Osterrieth, 1944), participants are asked to copy a complex figure in as much detail as possible. After a 30-min delay, participants are asked to redraw the complex figure from memory in as much detail as they can.

### Magnetic resonance imaging (MRI) scanning protocol

Structural MRI scans were obtained at the Montreal Neurological Institute with a 1.5T Siemens Sonata MRI scanner. Participants were comfortably placed in the scanner with their heads immobilized using an air cushion. An anatomical scan of ~15 min was taken for each participant. A 3D gradient echo acquisition is used to collect 160 contiguous 1 mm T1–weighted images in the sagittal plane (TR = 22, TE = 10, flip angle = 30°, 140 1 mm sagittal slices). The rectangular field of view (FOV) for the sagittal images is 256 mm (SI) by 224 mm (AP).

### MRI data analysis

VBM was used to investigate morphological differences between the different groups. MRI scans were spatially normalized by linear transformation into a standard stereotaxic Talairach space (Talairach and Tournoux, 1988). They were corrected for intensity non-uniformity (shading artifact) using the N3 software package (Sled et al., 1998). Each voxel was automatically labeled as white matter, gray matter, cerebrospinal fluid, or background using Intensity Normalized Stereotaxic Environment for the Classification of Tissues (INSECT) (Zijdenbos et al., 2002). The skull and dura were then masked from the brain. The gray matter was smoothed using a 8 mm full-width at half-maximum (FWHM) Gaussian kernel. Generalized linear model was used to correlate performance on the CSDLT with gray matter in our regions of interest, namely the hippocampus and caudate nucleus (Worsley et al., 2002). After this initial analysis, results were used to regress gray matter values from our peak voxel in the hippocampus against the whole brain. This analysis allowed us to examine areas in the brain that co-vary with gray matter in the hippocampus.

Outputs of the statistical analyses were displayed as a statistical map overlaid on an MRI scan. For results within the hippocampus, statistical maps were overlaid on an MRI scan of a spatial learner, categorized by performance on the recombined pairs condition. The statistical maps show regions of gray matter that correlate with our variable of interest. Based on our a priori hypothesis, an uncorrected *p*-value of 0.001 (*N* = 45, *t* = 3.29) was used for voxels in the predicted regions of interest, namely, the hippocampus and caudate nucleus. For the whole brain, a Bonferroni correction for multiple comparisons was used to calculate the *t*-statistical threshold (*t* = 5.47 at *p* < 0.05).

## Results

### Behavioral

All participants were able to learn the reward contingency of the arms during the encoding phase of the CSLDT. On average participants required 10.02 ± 4.15 trials to reach criteria. Average performance on Stage 2 was 5.55 ± 1.96 out of 8. On the RAVLT, participants were able to recall an average of 10.83 ± 2.76 of the 15 words AI and 10.37 ± 3.38 after the 30-min delay. After the 30-min delay of the RO, participants had an average score of 18.14 ± 6.83 out of 32. Performance on Stage 2 of the CSDLT did not correlate with performance on the RAVLT (AI: *r* = 0.012, *p* > 0.05; Delayed recall: *r* = 0.093, *p* > 0.05) or RO (*r* = −0.155, *p* > 0.05). There was also no correlation between performance on Stage 2 of the CSDLT and age (*r* = −0.061, *p* > 0.05), years of education (*r* = 0.019, *p* > 0.05), or sex (*t* = 1.19, *p* > 0.05).

### VBM

**Figure 2:**
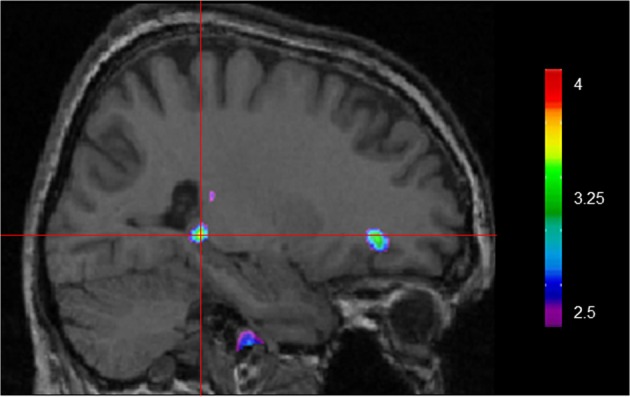
**Whole brain regression analysis with scores on recombined pairs condition of CSDLT (Stage 2).** Results are superimposed onto an anatomical MRI and displayed in the sagittal plane. Results show that gray matter in the hippocampus significantly co-vary with spatial navigational strategies (MNI space coordinates *x* = 26.0, *y* = −37.8, *z* = −3.2; *t* = 3.53, *p* < 0.0005).

**Figure 3:**
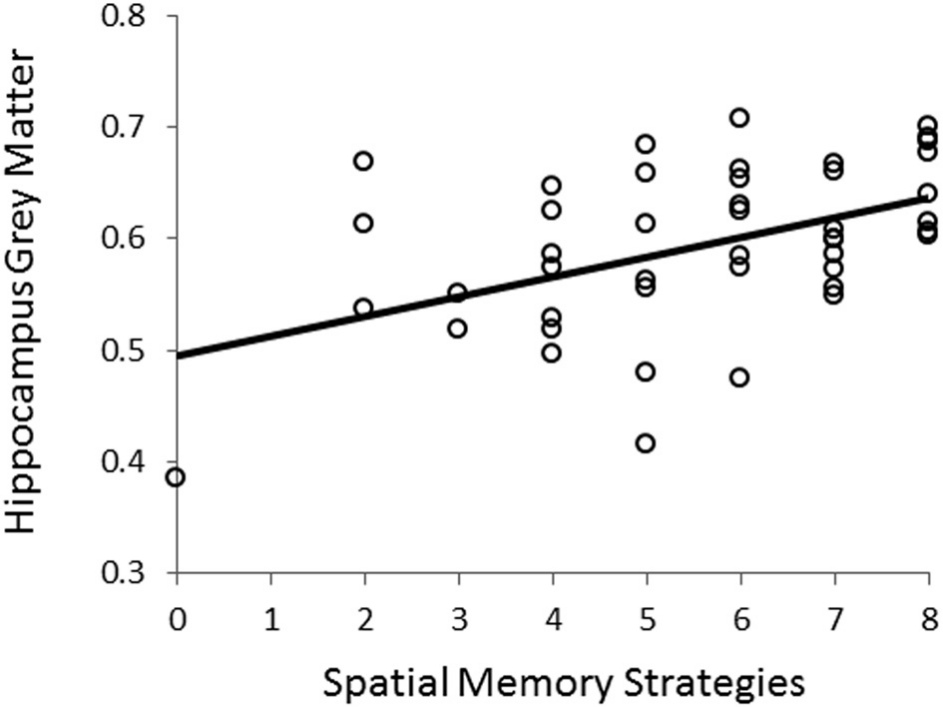
**Correlation between scores on the recombined pairs condition (Stage 2) of the CSDLT and gray matter values extracted from the peak voxel (MNI space coordinates *x* = 26.0, *y* = −37.8, *z* = −3.2) in the right posterior hippocampus (*r* = 0.478, *p* < 0.001).** This graph shows that a better spatial memory score is associated with increased gray matter in the hippocampus.

Using VBM, performance on the CSDLT was regressed against gray matter in the brain. Performance during the recombined pairs condition (Stage 2) of the CSDLT positively correlated with gray matter in the right hippocampus (MNI space coordinates *x* = 26.0, *y* = −37.8, *z* = −3.2; *t* = 3.53, *p* < 0.0005), suggesting that healthy older adults who have higher scores on the recombined pairs condition and thus use spatial strategies, have more gray matter in the hippocampus (Figure 2). The correlation between the peak gray matter value in the hippocampus and performance on the recombined pairs condition of the CSDLT was *r* = 0.478, *p* < 0.001 (Figure 3). No areas of the brain, including the caudate nucleus, were found to negatively correlate with the performance on the recombined pairs condition of the CSDLT. In other words, we did not find a correlation between low score on the recombined pairs condition, indicative of a response strategy and gray matter in the caudate nucleus (*p* > 0.001, uncorrected). The number of trials to reach criteria did not correlate with gray matter in the hippocampus or caudate nucleus.

**Figure 4:**
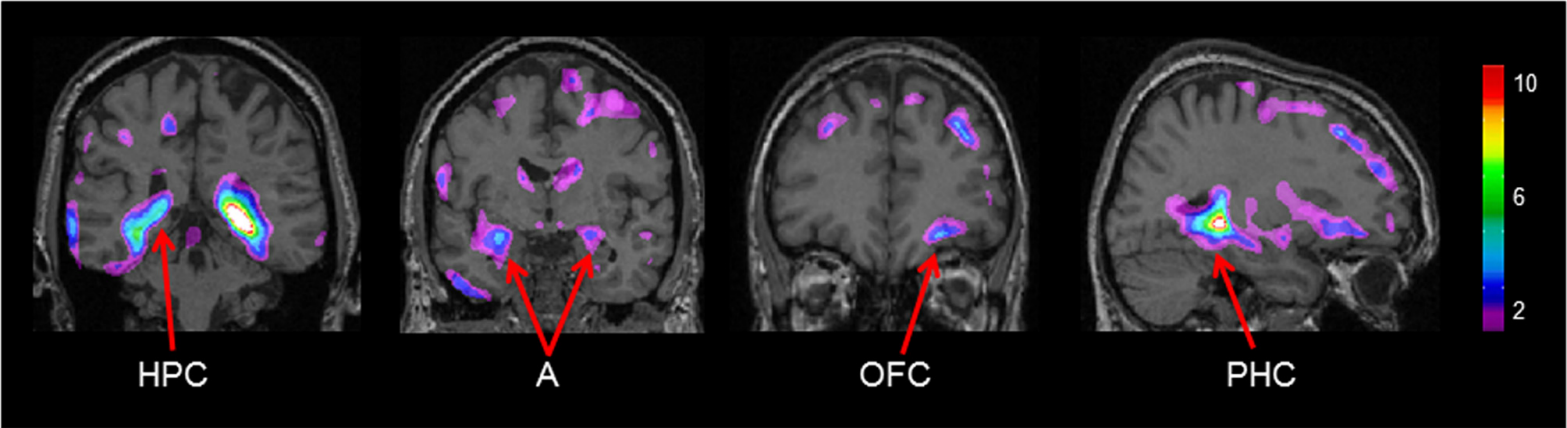
**Regions of the brain that co-varied with our peak hippocampal gray matter value (*x* = 26.0, *y* = −37.8, *z* = −3.2).** A, Amygdala; HPC, hippocampus; PHC, parahippocampal cortex; OFC, orbitofrontal cortex. The color bar illustrates the range of *t* statistical values.

Gray matter values extracted from our peak voxel in the hippocampus (from the first analysis) were used to regress against the entire MRI volume in all participants. Results showed a network of regions known to be anatomically linked to the hippocampus co-varying with the peak voxel in the right hippocampus (Figure 4). Consistent with our earlier findings (Bohbot et al., 2007), with increasing gray matter in the hippocampus, there was increased gray matter in the contralateral hippocampus, the right orbitofrontal cortex, bilateral amygdala, and bilateral parahippocampal cortex.

### Neuroanatomical correlates of neuropsychological measures

Performance AI and delayed recall on the RAVLT did not correlate with hippocampus gray matter. Similarly, delayed recall on the RO did not correlate with gray matter in the hippocampus suggesting that spatial memory is more sensitive to gray matter differences in the hippocampus than other types of memory measured with standard Neuropsychological tests.

## Discussion

In the present study, we examined the relationship between performance on a virtual navigation task and gray matter in the hippocampus in healthy older adults. With VBM, we showed that the spontaneous use of spatial memory strategies positively correlates with gray matter in the hippocampus. In contrast, the use of response strategies negatively correlates with gray matter in the hippocampus. When the peak value in the hippocampus, the area in the hippocampus which most highly correlated with spatial memory, was used as a seed voxel to correlate with gray matter in the whole brain, positive correlations were observed in regions that are anatomically and functionally linked to the hippocampus, such as the contralateral hippocampus, the right orbitofrontal cortex, bilateral amygdala, and bilateral parahippocampal cortex.

While some studies in the literature reported a significant relationship between hippocampal volume and spatial memory (Chen et al., 2010; Head and Isom, 2010), others have failed to demonstrate this effect (Driscoll et al., 2003; Moffat et al., 2007). One factor that may help shed light on this issue concerns the fact that none of these studies involved tasks that assessed spontaneous navigational strategies (when both spatial and response strategies can be used in the same task). Indeed, ours is the first structural MRI study to utilize a task in older adults that distinguishes between navigational strategies. This is a notable distinction because both human and non-human animal studies showed that with aging, there is a decreased use of spatial strategies and increased use of response strategies (Barnes et al., 1980; Rapp et al., 1997; Rodgers et al., 2010; Etchamendy et al., 2011). Therefore, older adults tested on the virtual Water Maze (e.g., Moffat et al., 2007) may have performed well on that task despite the fact that they would have used a response strategy dependent on the caudate nucleus when tested on another task allowing for both strategies (Etchamendy and Bohbot, 2007). If this is the case, an association between task performance and hippocampal volume would not necessarily be found. On the other hand, as mentioned above, some spatial memory tasks that did not measure spontaneous navigational strategies were sensitive to gray matter in the hippocampus (Chen et al., 2010; Head and Isom, 2010). Further research would be needed in order to shed light on this issue. For instance, some of these spatial tasks may have been sufficiently complex to detect a learning deficit in participants favoring response strategies, thereby inadvertently dissociating between spatial and response strategies. In support of this hypothesis, we previously reported a correlation between spatial strategies and wayfinding performance in healthy older adults (Etchamendy et al., 2012). This would be consistent with our results that demonstrate that in fact only older adults using spatial strategies, and not those using response strategies, have more gray matter in the hippocampus.

The study by Head and Isom (2010) reported a correlation between caudate nucleus volume and route learning. Route learning is a form of response learning that involves learning a sequence of movements in response to specific stimuli. However, we did not observe a negative correlation between performance and caudate nucleus gray matter with the current task, suggesting that it may not be sensitive enough to detect variability in the gray matter of the caudate nucleus of our population. Interestingly, Kennedy et al. (2009) demonstrated a non-linear decline in hippocampus volume as a function of age, where the rate of hippocampus atrophy accelerates with age, while Raz et al. (2003) showed that in the caudate nucleus, there is a linear decline in volume with aging. Therefore, within an older adult population, there may be more variability in the volume of the hippocampus than in the volume of the caudate nucleus, making it easier to detect morphological differences within the hippocampus. Recruiting participants in a wider age range may help increase sensitivity to morphological differences in the caudate nucleus. Indeed, the age range of the participants in the Head and Isom (2010) study was 56–86 years, while in the current study it was 60–75 years. Therefore, a wider age range of participants may be needed to uncover the relationship between gray matter in the caudate nucleus and response strategies in our task.

We observed an association between the aging hippocampus and response strategies. However, the causal relationship cannot be determined in this study, i.e., the use of response strategies may be the consequence of decreased gray matter in the hippocampus or it may be the cause of a decrease gray matter in the hippocampus, i.e., “use it or loose it.” In other words, biological factors such as genes (Banner et al., 2011) may have a negative impact on the hippocampus leading to an increase use in response strategies. Alternatively, environmental factors such as repetition (which leads to habit) (Iaria et al., 2003), stress (Schwabe et al., 2008), or reward (Del Balso et al., 2010) could promote response strategies at the expense of spatial strategies associated with the hippocampus. It is most likely a combination of both. Importantly, the fact that we found an association between response strategies and the aging hippocampus opens new possibilities for spatial memory-based cognitive interventions toward healthy aging (Bohbot et al., 2011). We have previously shown that strategy use can alter gray matter in the mouse hippocampus (Lerch et al., 2011). In that study, mice trained to use spatial strategies showed increased gray matter in the hippocampus relative to mice trained on the response strategy. Preliminary findings in older adults replicated these findings, demonstrating increased gray matter in the hippocampus after the administration of spatial memory training (Fouquet et al., 2011).

The relationship between navigational strategy and the hippocampus in young adults was previously investigated in Iaria et al. (2003) and Bohbot et al. (2007). Iaria et al. (2003) found that in a task where both strategies can be used, 50% of young adults spontaneously used a spatial strategy while 50% used a response strategy. Furthermore, individuals who used a spatial strategy showed significant fMRI activity in the hippocampus, while those who used a response strategy showed significant fMRI activity in the caudate nucleus, relative to baseline. Bohbot et al. (2007) found that the spontaneous use of spatial strategies was associated with increased gray matter in the hippocampus. The same technique of VBM was used to analyze the results in both Bohbot et al. (2007) and the current study. However, a different navigation task was used to assess navigational strategy. In Bohbot et al. (2007), the 4-on-8 virtual maze task was used while in the current study the CSDLT was used. Consistent results were obtained in the two studies.

Similar to Bohbot et al. (2007), a number of brain areas anatomically linked to the hippocampus correlated with gray matter values extracted from our peak voxel in the hippocampus in older adults, such as the orbitofrontal cortex, the parahippocampal cortex, and the amygdala. It is possible that the co-activity of this network of regions during navigation leads to increased gray matter. It has been suggested that the orbitofrontal cortex in rodents is involved in spatial memory (Vafaei and Rashidy-Pour, 2004) and learning reward expectancy in a spatial context (Young and Shapiro, 2011). Indeed, orbitofrontal cortex neurons respond when a rodent enters an arm that is expected to contain a reward. The parahippocampal cortex is known to be involved in navigation and its functions involve scene processing and spatial learning (Bohbot et al., 1998; Epstein, 2008). In rodents, the amygdala is associated with the hippocampus in place preference learning when it is associated with reward (Gaskin and White, 2006).

We also investigated the relationship between neuropsychological performance and gray matter in the hippocampus. Previous studies have found a correlation between the RAVLT and volume of the left hippocampus (Hackert et al., 2002; Chen et al., 2010), however we did not find this effect in our study. The inconsistency from the literature may arise from a cohort effect. The current sample population is composed of high functioning older adults with no detectable cognitive impairment. The RAVLT and RO may therefore not be sensitive enough to detect differences in gray matter in a more homogeneous population. These results suggest that spatial memory strategies are more sensitive to structural differences in the hippocampus than standard neuropsychological tests. In fact, a prospective study showed that patients with dementia had deficits in spatial cognition that preceded conversion to dementia by 3 years. In the same cohort, verbal memory, and working memory deficits were detected only 1 year before patients were diagnosed with dementia (Johnson et al., 2009), further demonstrating the sensitivity of spatial memory.

In conclusion, our present findings are consistent with the literature reporting that in healthy older adults there is a positive correlation between spatial memory and gray matter in the hippocampus. Furthermore, our results showed that those who have lower hippocampal gray matter are actually using another form of navigational strategy, the response strategy. With whole brain analysis, we observed that individuals with more gray matter in the hippocampus had more gray matter in anatomically and functionally connected cortical areas, namely the contralateral hippocampus, the right orbitofrontal cortex, bilateral amygdala, and bilateral parahippocampal cortex. Future studies will be needed to examine whether an intervention method focusing on spatial memory training can increase gray matter in the hippocampus and anatomically connected areas. Promoting the use of spatial strategies can be a potential avenue for intervention methods against hippocampal atrophy (Fotuhi et al., 2012). As lower hippocampal volume is a risk factor for cognitive decline as well as a number of disorders including Alzheimer's disease (Convit et al., 1997; Rusinek et al., 2003; Tapiola et al., 2008), promoting the use of spatial memory can potentially have protective effects against cognitive deficits in normal aging as well as risks of degenerative disorders of the hippocampus.

### Conflict of interest statement

The authors declare that the research was conducted in the absence of any commercial or financial relationships that could be construed as a potential conflict of interest.
